# Disease burden metrics and the innovations of leading pharmaceutical companies: a global and regional comparative study

**DOI:** 10.1186/s12992-020-00610-2

**Published:** 2020-09-10

**Authors:** Ye Lim Jung, JeeNa Hwang, Hyoung Sun Yoo

**Affiliations:** 1 Division of Data Analysis, Korea Institute of Science and Technology Information (KISTI), Seoul, 02456 Republic of Korea; 2 Science and Technology Management Policy, University of Science and Technology, 217 Gajeong-ro, Yuseong-gu, Daejeon, 34113 Republic of Korea

**Keywords:** Pharmaceutical innovation, Innovation activities, Disease burden, Multinational pharmaceutical companies, Public-private partnerships

## Abstract

**Background:**

The recent innovation activities of global top-tier pharmaceutical companies in accordance with global and regional health concerns were investigated in order to identify their innovations contributing to population health.

**Methods:**

“Innovation activity” was defined as the number of drugs for which R&D activities have been reported within the last three years. Such activities were measured by collecting the data on drug developments and classifying them by developer company, phase of development, therapeutic use, and the country in which the development conducted. Subsequently, we examined and compared the correlations between the global innovation activities of the top 20 pharmaceutical companies and the disease burden measured in disability-adjusted life years (DALYs) by income level and region. In addition, this study analyzed the association between country-specific innovations and DALYs in the corresponding countries.

**Results:**

At a global level, the innovation activities were not associated with global DALYs. However, when analyzed by income level, the innovation activities were associated with DALYs in high income and upper middle income countries while it was not associated with DALYs in low middle income and low income countries. In terms of region, correlations were found between the innovation activities and DALYs in the European region, the Americas, and the Western Pacific region whereas such correlations were not found in the African, Eastern Mediterranean, and South-East Asian regions. Similar to the analyses by income level and region, correlations between country-specific innovations and DALYs were only found in high income or high GDP countries. In addition, an empirical analysis of several cases including Canada, Germany, South Korea, and the United Kingdom revealed that pharmaceutical innovation is more closely related to market size than disease burden.

**Conclusions:**

This study identified that discrepancies between pharmaceutical innovation and public health needs, i.e., disease burden values, have persisted until recently. To alleviate this imbalance, both public and private sectors should not only fulfill their respective roles and responsibilities regarding these issues, but also make strategic and collaborative efforts such as Product Development Partnerships (PDPs) directed toward public health improvement.

## Introduction

The pharmaceutical industry has a unique characteristic that differentiates it from other industries, namely the fact that it directly impacts human lives and public health [1]. Humanity’s universal and strong desire for healthy conditions and prolonged lifespans makes the pharmaceutical industry indispensable and attractive, creating constant demand. For these reasons, the pharmaceutical industry formed a huge market, globally worth 1.2 trillion US dollars (USD) in 2018, with an annual growth rate of 6.3% over the last five years [2]. Another unique characteristic of the pharmaceutical industry is that it is one of the industries that invest most heavily in research and development (R&D) [3]. The health industry, including pharmaceuticals, had the second largest scale of R&D, following the ICT industry [4, 5]. This may be mainly attributed to the difficulties that are involved in drug discovery and development, such as long periods of lead/candidate identification, time-consuming process of clinical trials, high development cost per product, and extremely low rate of successful outcomes [1].

In this regard, new product developments by pharmaceutical companies tend to focus primarily on profitable drugs, to compensate for the companies’ tremendous investments and efforts devoted to the arduous process of drug development [6]. This is reasonable and only to be expected, given that the ultimate goal of private companies is to maximize their financial return. However, this profit motive is not always in optimal accordance with demands from a public health perspective. That is, the drugs that pharmaceutical companies prefer to develop may not be adequately aligned with global public health needs [7, 8], even though these companies have significantly contributed to alleviating disease burdens and have resolved many global health challenges [9, 10].

Considerable efforts have been made to investigate the imbalance between pharmaceutical innovation and public health demands [11–16]. Previous studies have revealed areas that lack adequate drug developments, requiring governmental and policy-based support. However, it is not enough to depend solely on the public sector to take responsibility for offering support or investment. With the growth of the pharmaceutical industry, there have been increasingly vocal demands that the public and private sectors should make an effort together for the improvement of global population health [17, 18]. Public-Private Partnerships (PPPs) are good examples of such cooperation, which can effectively bridge the gap between health needs and pharmaceutical innovation [19, 20].

Since many of the successful drug development projects are led by large pharmaceutical companies, there is a particularly more need for the active participation of such large firms in collaborative efforts. Although the roles of small and emerging biopharmaceutical companies are rising in significance [21], most drugs on the market are still dominated by several multinational companies. Since they generate immense revenues from businesses all over the world, these firms can better afford to invest sufficiently in R&D and build a diverse product portfolio. Consequently, their R&D should further contribute to reducing the disease burden from public health interests, not only in pursuit of corporate profit [22, 23].

In this context, we investigated the drug development activities of global top-tier pharmaceutical companies in accordance with global and regional health concerns, in order to identify how their innovations contribute to population health. Specifically, we analyzed the relationship between the pharmaceutical innovations of the top 20 leading global companies and global, regional, and country-specific disease burdens, measured by disability-adjusted life years (DALYs). Our study found clearer evidence than had been available in previous studies of the tendency of innovation activities to be discriminatory by income level or region. We also pinpointed the diseases or countries that have been left behind in the innovation. Finally, we discussed implications of these findings for both private and public sectors, regarding the goal of public health improvement.

## Methods

### Data collection and refinement

We defined “innovation activities” as the number of drugs for which R&D activities have been reported within the last three years (from July 2016 to June 2019). In order to identify the status of the most recent innovation activities, drugs that were already released on the market three years ago and R&D activities prior to the last three years were excluded from the dataset. In addition, generic drugs and OTC (Over the Counter) drugs were also ruled out to identify genuinely innovative drugs. That is, we narrowed the scope of this study by focusing on the top 20 companies’ recent R&D activities, to investigate whether their direction of innovations is well aligned with needs from a public health perspective.

The data on the drugs which satisfied the above criteria were obtained from IQVIA™ Pipeline Intelligence [24]. This data source covers 170 countries worldwide. From 4156 pharmaceutical companies that were included in the above dataset, only the top 20 companies, whose global prescription drug sales in 2018 were over 15 billion USD [4], were included in our main analysis. This is because these leading companies can better afford to consider public health needs and have greater responsibility to do so, compared to small companies when building their products portfolio. The top 20 companies are Pfizer, Roche, Novartis, Johnson & Johnson, Merck & Co., Sanofi, AbbVie, GlaxoSmithKline, Amgen, Gilead Science, Bristol-Myers Squibb, AstraZeneca, Eli Lilly, Bayer, Novo Nordisk, Takeda, Celgene, Boehringer Ingelheim, Allergan, and Teva Pharmaceutical Industries. They accounted for 63.4% of worldwide prescription drug sales in 2018 [4].

The innovation activities were classified according to the Anatomical Classification (AC) system established by the European Pharmaceutical Market Research Association (EphMRA). In this classification system, drugs are classified according to their indications and therapeutic use [25]. In cases where a drug is assigned to more than one anatomical classes, we allowed multiple counting of such a drug with multiple AC codes, once for each class to which it belongs. The top 20 companies’ innovation activities by country were measured by extracting the number of cases of R&D performed at any phase of the drug development within a given country.

Global, regional, national and income level-specific DALYs in 2016 were obtained from the Global Burden of Disease (GBD) Study of the World Health Organization (WHO) [26]. The DALYs per 100,000 population were applied to our comparative analysis. The pharmaceutical sales in 2016 in Canada, Germany, South Korea, and the United Kingdom (UK) were obtained from the Organization for Economic Cooperation and Development (OECD) statistics [27].

### Data transformation and analysis

Since the innovation activities were measured according to the AC of EphMRA and the DALYs values were measured according to GBD’s disease classification (GBD causes), data conversion was required to impose a unified standard. Therefore, we constructed an AC-ICD-GBD mapping table to convert the innovation activities counted based on AC to data based on GBD causes. First, we performed AC-ICD matching by using the information regarding which ICD code was most prescribed for each AC code; this information was obtained from IQVIA™ therapeutic class profiles. Second, ICD-GBD matching was performed by using the list of GBD causes mapped to ICD 10 codes, acquired from the Institute for Health Metrics and Evaluation (IHME) [28]. In cases where the ICD prescription information by AC code was not available, direct matching from AC to GBD was conducted qualitatively using the expert’s domain knowledge. The mapping table was constructed using R software (version 3.5.3).

By utilizing the mapping table, the data on innovation activities were calculated based on GBD causes. Finally, the correlations between innovation activities and DALYs or estimated market size were analyzed by the Pearson correlation coefficient at a 95% confidence level and univariate linear regression analysis was also performed to investigate the relationship between them. All variables were converted to a natural logarithmic scale for analysis, except in the case of COVID-19 analysis. All analyses were carried out with the SPSS statistical package (version 20.0, Chicago, Ill, USA).

## Results

The number of drugs for which R&D activities were performed globally, in all pharmaceutical companies over the last three years (from July 2016 to June 2019), was 10,550 (Table 1). Analyzing by phase, we observed that the highest number of these drugs were in the preclinical (28.2%) stage, phase II (18.4%) and phase I (17.0%) stages. About half of the total drugs were biological products (46.5%) and 10.2% of the total were the drugs designated as orphan drugs.

**Table 1:** Summarized overview of global innovation activities on drug development in pharmaceutical companies (July 2016 – June 2019)

	All pharmaceutical companies (Number of drugs, (%))	Top 20 leading companies (Number of drugs, (%))	Percentage of top 20 companies
Total	10,550 (100)	2669 (100)	25.3%
Phase			
Discovery	1406 (13.3)	260 (9.7)	18.5%
Preclinical	2979 (28.2)	275 (10.3)	9.2%
Clinicals	144 (1.4)	7 (0.3)	4.9%
Phase I	1794 (17)	523 (19.6)	29.2%
Phase II	1936 (18.4)	482 (18.1)	24.9%
Phase III	817 (7.7)	230 (8.6)	28.2%
Pre-registration	168 (1.6)	46 (1.7)	27.4%
Registered	146 (1.4)	37 (1.4)	25.3%
Marketed	1070 (10.1)	754 (28.3)	70.5%
N/A (Technology)	90 (0.9)	55 (2.1)	61.1%
Orphan drugs^a^	1076 (10.2)	355 (13.3)	33.0%
Biologic products^b^	4905 (46.5)	1165 (43.6)	23.8%

^a^ Drugs being developed for rare diseases and have been designated as orphan drugs

^b^ Drugs being developed as biologic therapies

The top 20 companies accounted for 25.3% of total drugs in all pharmaceutical companies (Table 1). Noticeably, they possessed at least more than 24.9% of total drugs in each stage above Phase I and possessed as many as 70.5% of the marketed drugs. When innovations were counted by AC, we found that antineoplastic and immunomodulating agents (the L class) possessed the largest number of drugs (31.4%) followed by the alimentary tract and metabolism (the A class) (12.6%), and the central nervous system (the N class) (11.4%) (Table 2). Parasitology (the P class) had the smallest number of drugs (0.6%) among them all.

**Table 2:** Top 20 leading pharmaceutical companies’ innovation activities by therapeutic use

Classification of therapeutic use^a^	Top 20 companies’ innovation activities^b^ (Number of drugs (%))
Alimentary tract and metabolism (A class)	404 (12.6)
Blood and blood forming organs (B class)	95 (3)
Cardiovascular system (C class)	151 (4.7)
Dermatologicals (D class)	144 (4.5)
Genito-urinary system and sex hormones (G class)	103 (3.2)
Systemic hormonal preparations (excl. Sex hormones) (H class)	21 (0.7)
General anti-infectives systemic (J class)	286 (8.9)
Antineoplastic and immunomodulating agents (L class)	1008 (31.4)
Musculo-skeletal system (M class)	144 (4.5)
Central nervous system (N class)	366 (11.4)
Parasitology (P class)	19 (0.6)
Respiratory system (R class)	162 (5)
Sensory organs (S class)	84 (2.6)
Diagnostic agents (T class)	21 (0.7)
Various (V class)	207 (6.4)

^a^Classified based on the EPhMRA classification system

^b^Multiple counting was allowed for drugs which have several therapeutic uses, and there were assigned more than one class

In terms of DALYs measured by GBD causes, globally in 2016, cardiovascular diseases showed the highest DALYs (4476.9) followed by diarrhea, lower respiratory, and other common infectious diseases (3110.8) and neoplasms (2884.4) (Table S1 in Supplementary Information). By income level classification, neoplasms (4431.4) ranked first in high income countries whereas diarrhea, lower respiratory, and other common infectious diseases (9960.3) ranked first in low income countries. In terms of regional classification, cardiovascular diseases showed the highest DALYs in the Eastern Mediterranean Region (4650.9), the European Region (7369.0), and the Region of the Americas (3624.6), the South-East Asia Region (4871.7) and the Western Pacific Region (5314.9) while diarrhea, lower respiratory, and other common infectious diseases showed the highest DALYs in the African Region (9849.4).

The correlation analyses of the top 20 leading companies’ recent innovation activities with DALYs were performed by income level or regional categorization of the countries in the world (Table 3). At a global level, their innovation activities were not associated with global DALYs. However, once the countries were grouped according to the World Bank’s income level classification, we found that the innovation activities were associated with DALYs in high income (r = 0.617 (*p* = 0.008)) and upper middle income (r = 0.539 (*p* = 0.025)) countries. By contrast, the innovation activities were not associated with DALYs in low middle income and low income countries.

**Table 3:** Association of the top 20 leading companies’ global innovation activities with DALYs by income level and region

	Correlation coefficient (r)
Global	0.243
Income level^a^	
High Income	0.617^**^
Upper Middle Income	0.539^*^
Lower Middle Income	0.067
Low Income	−0.396
Region^b^	
European Region	0.596^*^
Western Pacific Region	0.557^*^
Region of the Americas	0.506^*^
South-East Asia Region	0.232
Eastern Mediterranean Region	0.161
African Region	−0.414

^a^Classified by the World Bank

^b^Classified by the WHO

^*^p < 0.05

^**^p < 0.01

In terms of the WHO regional classification, associations were found between the innovation activities and DALYs in the European region (r = 0.596 (*p* = 0.012)), the region of Americas (r = 0.506 (*p* = 0.038)), and the Western Pacific region (r = 0.557 (*p* = 0.020)). In contrast, the innovation activities were not associated with DALYs in the African, Eastern Mediterranean, and South-East Asia regions.

**Fig. 1:**
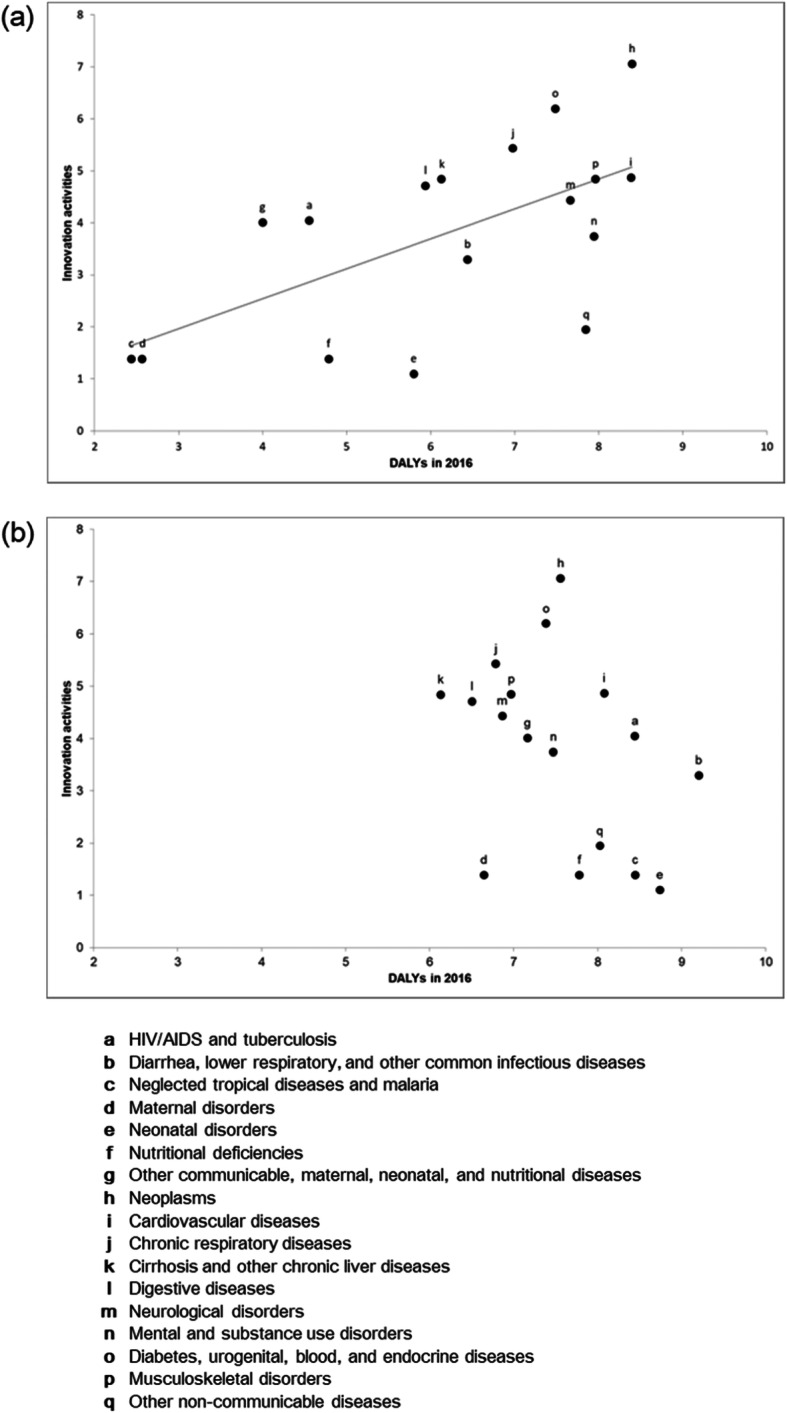
Relationship between the top 20 leading companies’ global innovation activities and DALYs in high income countries (**a**) and low income countries (**b**)

We compared the relationship between global innovation activities and DALYs in high income countries and low income countries (Fig. 1). In high income countries, although the innovation activities were highly associated with DALYs, significant discrepancies were observed for neoplasms (‘h’ in Fig. 1(a)), diabetes, urogenital, blood, and endocrine diseases (‘o’ in Fig. 1(a)), and other communicable, maternal, neonatal, and nutritional diseases (‘g’ in Fig. 1(a)), which showed a larger number of innovation activities than the expected values that had been obtained when the DALYs were applied as a predictor. Other non-communicable diseases (‘q’ in Fig. 1(a)), neonatal disorders (‘e’ in Fig. 1(a)), and nutritional deficiencies (‘f’ in Fig. 1(a)) showed a fewer number of innovation activities than the expected values. In low income countries, the DALYs values (per 100,000 population) for most diseases were much higher than those in high income countries, but no association was observed between the innovation activities and the DALYs (Fig. 1(b)).

We further explored the correlations between innovation activities and DALYs in several countries located in each region of the world (Table 4). For our comparative analysis by country, only the R&D activities that had taken place in a particular country were counted as country-specific innovations. Similar to the analyses of the global innovation activities and the DALYs by income level and region, associations between country-specific innovation and DALYs were found in high income or high GDP countries (Germany, Hungary, and the UK in the European region, China, Japan, and South Korea in the Western Pacific region, and Canada, Uruguay, and the United States in the Region of the Americas). In contrast, the innovations were not associated with DALYs in the countries located in the South-East Asian (Bangladesh, Indonesia, and Myanmar), Eastern Mediterranean (Kuwait and Saudi Arabia), and African (South Africa and Uganda) regions. There were also countries that did not have any R&D activities for drug development over the past three years, such as Iran and Rwanda.

**Table 4:** Association of country-specific innovation activities with DALYs in the corresponding countries

Country	Correlation coefficient (r)
(European Region)	
Germany	0.649^**^
Hungary	0.670^**^
United Kingdom	0.563^*^
(Western Pacific Region)	
China	0.660^**^
Japan	0.659^**^
South Korea	0.633^**^
(Region of the Americas)	
Canada	0.641^**^
Uruguay	0.603^*^
United States	0.647^**^
(South-East Asia Region)	
Bangladesh	0.169
Indonesia	0.316
Myanmar	0.084
(Eastern Mediterranean Region)	
Iran	n/a
Kuwait	0.430
Saudi Arabia	0.346
(African Region)	
Rwanda	n/a
South Africa	0.311
Uganda	0.394

^*^p < 0.05

^**^p < 0.01

Additionally, we investigated the association between innovation activities and market size rather than DALYs. We empirically analyzed the cases of Canada, Germany, South Korea, and the UK where the relevant data were fully available [27]. Our correlation analysis of pharmaceutical sales and innovation activities by therapeutic category in each country showed that all countries have similar or higher levels of association between innovation activities and market size, compared to the correlation between innovation activities and DALYs in each country (Table 5).

**Table 5:** Pharmaceutical sales by disease category in Canada, Germany, South Korea, and the United Kingdom and their correlation with innovation activities in each country

Therapeutic category	Pharmaceutical Sales (2016, million USD)			
	Canada	Germany	South Korea	United Kingdom
Alimentary tract and metabolism	2884.1	4871.8	3438.8	2394.2
Blood and blood forming organs	802.9	3138.5	1919.8	1390.3
Cardiovascular system	2974.5	4830.1	2822.5	1728.5
Genito urinary system and sex hormones	1120.4	867.7	667.7	960.2
Systemic hormonal preparations, excluding sex hormones and insulins	376.0	1233.7	209.7	819.9
Antiinfectives for systemic use	1731.6	3582.1	2697	3168.8
Musculo-skeletal system	687.2	1554.3	1232.1	618.1
Nervous system	4231.6	5818.8	1931.9	4392.2
Respiratory system	1460.7	2360.9	969.2	2231.9
Associations with innovation activities in each country	0.706^*^	0.642	0.730^*^	0.783^*^

^*^p < 0.05

## Discussion

In this study, we investigated the relationship between the leading pharmaceutical companies’ global innovation activities, focusing on recent R&D activities, and DALYs by income level and region. In addition, the associations between country-specific innovation activities and DALYs in certain countries were further explored.

It was previously demonstrated that there has been a misalignment between big pharmaceutical companies’ research publications and the global burden of diseases [29]. The study indicated that the focus of the companies’ research did not match well with global health concerns. Our study investigated the research efforts of multinational pharmaceutical companies more in terms of practical productization than academic output, by analyzing the innovation activities performed in the drug development pipeline.

The results from our study identifying the disparity of innovations by income level and region are consistent with findings in previous studies that pharmaceutical innovations are biased toward developed countries [11, 12, 16]. We demonstrated this pattern with stronger evidence, by comparing the associations between innovations and DALYs, with the income levels of the countries divided into four groups and the regions of the world divided into six groups, with the most up-to-date dataset on reported R&D cases of the pharmaceutical companies. This disparity was observed not only in the case of the top 20 companies, but for all pharmaceutical companies that had R&D activities in the last three years, as shown in Table S2 in the Supplementary Material. This can be attributed to the fact that the top 20 companies’ innovation activities comprise a substantial proportion of total pharmaceutical innovation and other companies that are not included in the top 20 companies also prioritize the pursuit of business profit.

In regard to discrepancies by disease categories, this study confirmed that in high income countries, the innovation activities on neoplasms, diabetes, urogenital, blood, and endocrine diseases have continued to be excessive compared with their DALYs values until recently (Fig. 1(a)) [12, 16]. In low income countries, we found a severe scarcity of innovation activities for treating Communicable Diseases (CDs) such as diarrhea, lower respiratory diseases, other common infectious diseases, and neglected tropical diseases and malaria which have the highest burden values in low income countries (Fig. 1(b)).

Since most of the top 20 companies are located in the US or European countries, it is somewhat understandable that they may be more responsive to the disease burdens in these countries. However, in a world that is increasingly more globalized and closely connected, it may not be wise to focus R&D activities only on specific regions or countries. As in the cases of Middle East Respiratory Syndrome (MERS), Severe Acute Respiratory Syndrome (SARS), and Coronavirus disease 2019 (COVID-19), the outbreak of pandemics which began in developing countries can spread at any time to developed countries and pose a grave threat worldwide. In this regard, taking proactive innovation efforts that take into account diseases prevalent in underdeveloped countries can be beneficial not only to public health but also to corporations’ potential returns.

**Fig. 2:**
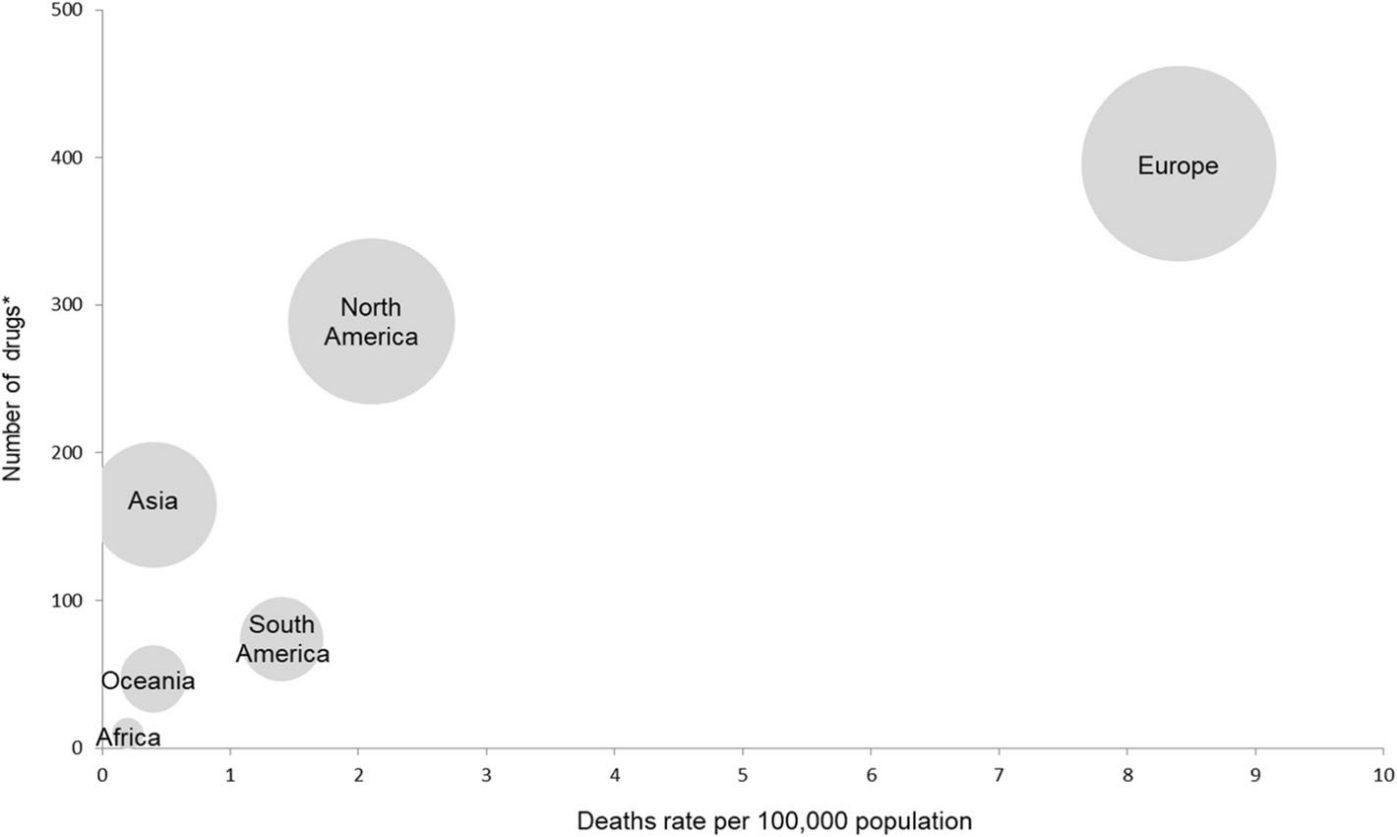
Comparison of the death rate (per 100,000 population) due to COVID-19 and the number of drugs being developed for COVID-19 by region (as of June 21, 2020). ^*^The size of the circle indicates the proportion of the drugs being developed in total

In the case of COVID-19, hundreds of thousands of people are losing their lives due to the unprecedented worldwide pandemic. To date (as of June 21, 2020), Europe and North America accounted for 61.6% of total confirmed cases and 80.1% of total deaths in the world [30]. Numerous pharmaceutical companies are making tremendous efforts to develop vaccines and treatments to combat COVID-19 and therefore 240 drugs are currently under development [24]. Since clinical trials on a single drug often proceed in several countries simultaneously, multiple counting by country was allowed, to investigate the drug development activities on COVID-19 by region. It was observed that drug development activities have been predominantly conducted in Europe (40.4%) and North America (29.5%) (Fig. 2). It is reasonable that R&D activities are biased toward these regions where developed countries are located, because the number of the confirmed cases and deaths due to COVID-19 are also predominantly high in these regions. On the other hand, it can be seen that the regions where low income countries are located such as Africa, Oceania, and South America are significantly marginalized in COVID-19 R&D activities.

However, the progression and prognosis of the virus can be influenced by race/ethnicity (e.g. immunological differences), climate, sociodemographic factors (e.g. population density and poverty rate), etc., which vary from region to region [31–36]. In addition, mutations of the virus, SARS-Cov-2, are continuously being reported as the spread of COVID-19 accelerates throughout the world [37, 38]. In this regard, targeting only specific countries or demographics would not be the most effective way to develop more potent vaccines and therapies. Moreover, in countries where healthcare resources are limited, timely and large-scale diagnosis, treatment, control, and prevention are all huge challenges that cannot be established in the near future, leading to national catastrophes. From this perspective, there is a strong need to consider marginalized regions and people together in both private and public sectors.

Several policy implications were derived from our study for both the private and public sectors. First, in the private sector, globally leading companies should further expand the development of drugs contributing to reducing the burden of marginalized diseases and/or countries, even if these types of drugs are less profitable. Of course, it should be acknowledged that the discrepancy between the innovation activities of the leading companies and DALYs is not particularly worse than the discrepancy found when we include the innovation activities of all other companies (Table S2 in Supplementary Information); furthermore, these leading companies should be given some credit for having contributed significantly to the development of orphan drugs. The top 20 companies accounted for 33% of the total innovation activities for orphan drugs during the period included in our analysis (Table 1). Over the past twelve years, multinational pharmaceutical companies’ investment in neglected tropical diseases (NTDs) has continuously grown, increasing fivefold since 2007, and they accounted for the vast majority (86%) of total industry sector investments in NTDs in 2018 [39]. However, despite these remarkable efforts and contributions, they accounted for only 16% of all funding for NTDs R&D in 2018 [39]. In other words, the investment in NTDs still depends heavily on the public sector. From this perspective, the leading companies which can better afford to manage their product portfolios taking into account both societal values and business returns, compared to small companies or startups where pursuit of profit is a top priority for survival, still have room for making meaningful progress in areas that have not fully benefited from support and investment for NTDs. Moreover, the public is increasingly demanding Corporate Social Responsibility (CSR) from large pharmaceutical companies [23]. It has been found that one of most preferred CSR activities by general public expected from pharmaceutical companies is the “promotion of public health” through activities such as the development of innovative drugs in untreated areas [40].

Secondly, the roles and responsibilities of the public sector remain crucial. The voluntary participation of private companies alone cannot sufficiently alleviate the disease burdens around the world. It has already been demonstrated that pharmaceutical innovation is significantly affected by market size [41, 42]. Our empirical analysis of several high income countries also proved that innovations are more closely related to market size than disease burden. In this context, it is natural that innovations are more concentrated in high income countries with large market sizes. Of course, diseases with high burden values are becoming more similar across both high and low income countries as non-communicable diseases (NCDs) have increased in low income countries in recent years [43]. Therefore, the drugs already developed for such diseases in high income countries can be delivered to low income countries to reduce the disease burdens in those countries. In particular, global leading companies have made striking contributions to ensure that these drugs are more widely available, accessible, and affordable in these countries, by utilizing diverse strategies such as equitable pricing, licensing, and product donations [9]. Their efforts and contributions should not be overlooked. However, even though the burden rankings of certain CDs such as HIV/AIDS, tuberculosis, diarrhea, malaria, and neonatal disorders have already been lowered in high income countries, they still rank high in low income countries (Table S1 in Supplementary Information). In addition, while major CDs such as HIV/AIDS, tuberculosis, and malaria are receiving high levels of R&D funding, other kinds of NTDs (WHO NTDs) have received very low levels of R&D funding, and what is worse, the funding levels have stagnated in place over the past decade [39]. There are still unresolved areas that require careful attention and further actions. In this regard, international non-profit institutions and non-governmental organizations (NGO) need to more extensively strengthen support and investment for these marginalized diseases and countries by accurately identifying the areas where drug development by the private sector itself has been scarce.

In addition, national governments should lead and foster basic research especially in areas where technological development is challenging due to the difficulties of mechanism identification or the lack of scientific knowledge. Such governmental initiatives should embrace a wide range of fundamental and original research conducted in various fields including academic, clinical, and industrial fields, which can contribute to population health in the long-term [44].

Finally, public and private sectors should cooperate strategically by utilizing collaborative programs such as Product Development Partnerships (PDPs) to develop products that are societally valuable but provide uncertain potential return, such as the vaccine for Acquired Immune Deficiency Syndrome (AIDS) [18, 45]. Specifically, the public sector should provide attractive incentives and benefits (e.g. assistance for clinical trials, ease of regulatory control, intellectual property protection, guaranteed profits, and so on) to the companies engaged in the programs. Since companies are fundamentally pursuing their own profit, it obviously to be expected that private firms will follow market forces. In this regard, the public sector can lead to increasing the market size of the disease areas where improvement is urgently required for the sake of public health. That is, governments can provide market incentives to induce the private sector to be more energetic in conducting innovation activities for diseases whose burden should be lowered more preferentially [46]. This can be accomplished by implementing policy strategies such as allowing companies to increase their drugs prices, expanding the coverage of national healthcare insurance, and compensating for the development costs and ensuring the profitability of the drugs. Several relevant policies have been implemented in many countries, but it is necessary to strengthen these policies for diseases with the highest burden globally.

The limitations of our study are as follows. First, when analyzing R&D activities by country, this study did not include cases where drugs that had already been developed elsewhere by leading pharmaceutical companies were delivered to marginalized countries through donation programs or free supplies, since we focused more on R&D activities than currently available drugs in a particular country. It should be recognized that billions of drugs and treatments are donated by the pharmaceutical industry partners. For instance, medicines for over 1.8 billion treatments were provided to impoverished and hardest-to-reach communities by donation programs in 2016 [47]. If further studies that broaden the scope beyond this study were to incorporate other types of innovations such as product donations, building/strengthening healthcare systems, education/training of clinicians and scientists, improving awareness of diseases, etc. in addition to the R&D pipeline analysis, such studies can be expected to yield richer and broader implications from diverse viewpoints. Secondly, there were some data losses in the process of data transformation. The innovation activities aggregated based on AC were sequentially converted to data based on ICD, followed by GBD causes. In the process, since the AC code was linked only to the top ICD code, the one that was the most prescribed, other diseases for which the drug was also prescribed were excluded from our analysis. Thirdly, other variables besides disease burden and market size that can affect pharmaceutical innovation could not be fully covered in this study due to the difficulties of acquiring relevant data. For example, by considering market concentration and/or degree of competition as other explanatory variables [48], the effects of each variable on pharmaceutical innovation could be investigated in more detail.

## Conclusions

The recent innovation activities of leading global pharmaceutical companies were comprehensively investigated by comparing differences in innovation by income level, region, and country. This study identified that there have not been substantial improvements in the imbalances and discrimination of pharmaceutical innovations, even though many concerns on this issue have been raised by researchers, health professionals, and healthcare decision makers. Filling the gap remains a challenging task and requires the cooperation of diverse stakeholders. In this regard, decision makers, policymakers, and pharmaceutical industry leaders ought to take stronger initiative to adopt and reference the implications and strategies derived from quantitative data analyses, such as the findings presented in this study, when setting public investment priorities, establishing health policies, and planning new businesses.

## Supplementary information

**Additional file 1: Table S1.** Global DALYs and DALYs by income level and region in 2016. **Table S2.** Association of the pharmaceutical companies’ innovation activities with DALYs by income level and region.

## Data Availability

The datasets supporting the conclusions of this article are available from the authors on reasonable request.

## Abbreviations

DALYs: Disability-Adjusted Life Years
GDP: Gross Domestic Product
USD: United States Dollar
R&D: Research and Development
ICT: Information & Communication Technology
PPPs: Public-Private Partnerships
OTC: Over the Counter
AC: Anatomical Classification
EphMRA: European Pharmaceutical Market Research Association
GBD: Global Burden of Disease
OECD: Organization for Economic Cooperation and Development
WHO: World Health Organization
ICD: International Classification of Diseases
IHME: Institute for Health Metrics and Evaluation
CDs: Communicable Diseases
MERS: Middle East Respiratory Syndrome
SARS: Severe Acute Respiratory Syndrome
COVID-19: Coronavirus Disease 2019
NTDs: Neglected Tropical Diseases
CSR: Corporate Social Responsibility
NGO: Non-Governmental Organizations
PDPs: Product Development Partnerships
AIDS: Acquired Immune Deficiency Syndrome
