# Supplementary material for: Disease burden metrics and the innovations of leading pharmaceutical companies: a global and regional comparative study
Source: Global Health. 2020 Sep 10;16:80. doi: 10.1186/s12992-020-00610-2 (PMC7481343; doi:10.1186/s12992-020-00610-2)
Supplement: Supplementary file 1 — Additional file 1: Table S1. Global DALYs and DALYs by income level and region in 2016. Table S2. Association of the pharmaceutical companies’ innovation activities with DALYs by income level and region. [file 12992_2020_610_MOESM1_ESM.docx]

Table S1. Global DALYs and DALYs by income level and region in 2016^a^

| Disease | Global | High Income | Upper Middle Income | Lower Middle Income | Low Income | African Region | Eastern Mediterranean Region | European Region | Region of the Americas | South-East Asia Region | Western Pacific Region |
| --- | --- | --- | --- | --- | --- | --- | --- | --- | --- | --- | --- |
| HIV/AIDS and tuberculosis | 1368.1 (10) | 94.2 (14) | 724.7 (11) | 1700.8 (8) | 4650.6 (4) | 5914.9 (3) | 616.7 (12) | 300.8 (13) | 313.5 (13) | 1325.2 (10) | 308.4 (13) |
| Diarrhea, lower respiratory, and other common infectious diseases | 3110.8 (2) | 622.8 (9) | 888.2 (9) | 4497.5 (2) | 9960.3 (1) | 9849.4 (1) | 3359.8 (3) | 880.4 (9) | 1093.8 (8) | 3814.9 (2) | 825.1 (9) |
| Neglected tropical diseases and malaria | 1014.5 (12) | 10.5 (17) | 147.4 (15) | 1355 (11) | 4657.5 (3) | 5822.7 (4) | 309.5 (16) | 13.2 (17) | 112.4 (15) | 494.3 (15) | 178.4 (14) |
| Maternal disorders | 186.2 (17) | 12.1 (16) | 33.7 (17) | 253.9 (17) | 771.9 (15) | 659.3 (15) | 271.5 (17) | 14.4 (16) | 57 (17) | 210.2 (17) | 24.1 (17) |
| Neonatal disorders | 2212.7 (5) | 328.9 (12) | 819.3 (10) | 3268.5 (3) | 6239.7 (2) | 6408.4 (2) | 3598.9 (2) | 569.4 (11) | 940.9 (10) | 2542.5 (4) | 607.9 (10) |
| Nutritional deficiencies | 824.3 (13) | 119 (13) | 236.7 (14) | 1263.9 (12) | 2388.8 (7) | 2234.9 (7) | 803.2 (11) | 195.5 (14) | 277.7 (14) | 1302.1 (11) | 175.7 (15) |
| Other communicable, maternal, neonatal, and nutritional diseases | 317.4 (16) | 53.6 (15) | 92.6 (16) | 400.2 (16) | 1292.7 (11) | 1047 (12) | 448.5 (14) | 68.8 (15) | 86.2 (16) | 351.9 (16) | 86.9 (16) |
| Neoplasms | 2884.4 (3) | 4431.4 (1) | 3595.3 (2) | 1876.6 (7) | 1918.5 (8) | 1657.1 (9) | 1808.7 (7) | 4491.2 (2) | 3069.2 (2) | 1891.3 (8) | 4061.9 (2) |
| Cardiovascular diseases | 4776.9 (1) | 4371.4 (2) | 5441.7 (1) | 4704.6 (1) | 3231.1 (5) | 2512.3 (6) | 4650.9 (1) | 7369 (1) | 3624.6 (1) | 4871.7 (1) | 5314.9 (1) |
| Chronic respiratory diseases | 1251.7 (11) | 1073.7 (8) | 1068.3 (8) | 1560.6 (10) | 886.9 (14) | 704.2 (14) | 888.5 (10) | 903.9 (8) | 973.7 (9) | 1970.7 (6) | 1254.3 (8) |
| Cirrhosis and other chronic liver diseases | 525.6 (14) | 457.9 (10) | 448.9 (12) | 633.2 (14) | 458.9 (17) | 467.5 (17) | 535.9 (13) | 687 (10) | 549.2 (11) | 602.6 (13) | 376.5 (11) |
| Digestive diseases | 464.9 (15) | 379.1 (11) | 394.6 (13) | 514.6 (15) | 667.8 (16) | 586.8 (16) | 377.1 (15) | 497 (12) | 431.8 (12) | 540.7 (14) | 352.3 (12) |
| Neurological disorders | 1401.2 (9) | 2125.9 (6) | 1365.3 (7) | 1244.4 (13) | 959.4 (13) | 972.7 (13) | 1329.3 (9) | 2035.6 (6) | 1630.4 (7) | 1285 (12) | 1342.4 (7) |
| Mental and substance use disorders | 2198.4 (6) | 2810 (4) | 2338 (4) | 1934 (5) | 1760.4 (9) | 1762 (8) | 2234 (5) | 2703.1 (3) | 2758.8 (3) | 1935.7 (7) | 2146.3 (4) |
| Diabetes, urogenital, blood, and endocrine diseases | 1809.3 (8) | 1776.8 (7) | 1739 (6) | 1925.2 (6) | 1612.6 (10) | 1646.2 (10) | 2016.2 (6) | 1535.4 (7) | 2146.1 (6) | 1983.9 (5) | 1577.1 (6) |
| Musculoskeletal disorders | 1894.3 (7) | 2862.9 (3) | 1999.7 (5) | 1604.5 (9) | 1066.1 (12) | 1095 (11) | 1695.8 (8) | 2663.2 (4) | 2208.5 (5) | 1698.7 (9) | 2047.9 (5) |
| Other non-communicable diseases | 2651.9 (4) | 2555.8 (5) | 2428.3 (3) | 2790.5 (4) | 3068.4 (6) | 3130.4 (5) | 3046.8 (4) | 2659.9 (5) | 2648.2 (4) | 2616 (3) | 2290 (3) |
| Total all causes | 33156.5 | 27787.2 | 27885.3 | 36238.8 | 49802.7 | 50109.8 | 33825.5 | 31956.7 | 27762.3 | 33617.8 | 26840.9 |

^a^DALYs per 100,000 population (rank).

Table S2. Association of the pharmaceutical companies’^a^ innovation activities with DALYs by income level and region

|  | | Correlation  coefficient (r) |
| --- | --- | --- |
| Global | | 0.320 |
| Income level^b^ | High Income | 0.696^**^ |
|  | Upper Middle Income | 0.602^*^ |
|  | Lower Middle Income | 0.148 |
|  | Low Income | -0.333 |
| Region^c^ | European Region | 0.673^**^ |
|  | Western Pacific Region | 0.620^**^ |
|  | Region of the Americas | 0.587^*^ |
|  | South-East Asia Region | 0.320 |
|  | Eastern Mediterranean Region | 0.278 |
|  | African Region | -0.355 |

^a^4,156 pharmaceutical companies that R&D activities are reported around the world are included.

^b^Classified by the World Bank.

^c^Classified by the WHO.

^*^*p* < 0.05.

^**^*p* < 0.01.
